# The value of maintaining normokalaemia and enabling RAASi therapy in chronic kidney disease

**DOI:** 10.1186/s12882-019-1228-y

**Published:** 2019-01-31

**Authors:** Marc Evans, Eirini Palaka, Hans Furuland, Hayley Bennett, Cecilia Linde, Lei Qin, Phil McEwan, Ameet Bakhai

**Affiliations:** 1 Diabetes Resource Centre, Llandough Hospital, Cardiff, UK; 2 Global Health Economics, AstraZeneca, Cambridge, UK; 3 Department of Nephrology, Uppsala University Hospital, Uppsala, Sweden; 4 Health Economics and Outcomes Research Ltd, Cardiff, UK; 5 Heart and Vascular Theme, Karolinska University Hospital and Karolinska Instiutet, Stockholm, Sweden; 6 Global Health Economics, AstraZeneca, Gaithersburg, MD USA; 7 School of Human and Health Sciences, Swansea University, Swansea, UK; 8 Department of Cardiology, Royal Free Hospital, London, UK

**Keywords:** Chronic kidney disease, Potassium, Hyperkalaemia, Renin-angiotensin-aldosterone system inhibitor, Economic modelling

## Abstract

**Background:**

People with chronic kidney disease (CKD) are at an increased risk of developing hyperkalaemia due to their declining kidney function. In addition, these patients are often required to reduce or discontinue guideline-recommended renin-angiotensin-aldosterone system inhibitor (RAASi) therapy due to increased risk of hyperkalaemia. This original research developed a model to quantify the health and economic benefits of maintaining normokalaemia and enabling optimal RAASi therapy in patients with CKD.

**Methods:**

A patient-level simulation model was designed to fully characterise the natural history of CKD over a lifetime horizon, and predict the associations between serum potassium levels, RAASi use and long-term outcomes based on published literature. The clinical and economic benefits of maintaining sustained potassium levels and therefore avoiding RAASi discontinuation in CKD patients were demonstrated using illustrative, sensitivity and scenario analyses.

**Results:**

Internal and external validation exercises confirmed the predictive capability of the model. Sustained potassium management and ongoing RAASi therapy were associated with longer life expectancy (+ 2.36 years), delayed onset of end stage renal disease (+ 5.4 years), quality-adjusted life-year gains (+ 1.02 QALYs), cost savings (£3135) and associated net monetary benefit (£23,446 at £20,000 per QALY gained) compared to an absence of RAASi to prevent hyperkalaemia.

**Conclusion:**

This model represents a novel approach to predicting the long-term benefits of maintaining normokalaemia and enabling optimal RAASi therapy in patients with CKD, irrespective of the strategy used to achieve this target, which may support decision making in healthcare.

**Electronic supplementary material:**

The online version of this article (10.1186/s12882-019-1228-y) contains supplementary material, which is available to authorized users.

## Background

Chronic kidney disease (CKD) is defined as a condition that impairs kidney function, causing kidney damage to worsen over several months or years. It can progress to end stage renal disease (ESRD), which is fatal without dialysis or kidney transplantation. International guidelines recommend renin-angiotensin-aldosterone system inhibitors (RAASi), such as angiotensin converting enzyme (ACE) inhibitors and angiotensin receptor blockers (ARBs), as first-line agents to prevent CKD progression [1, 2]. Treatment with RAASi has been shown to reduce blood pressure and proteinuria [3], delay estimated glomerular filtration rate (eGFR) decline [4], and lower the risks of kidney failure, cardiovascular (CV) morbidity and all-cause mortality in the CKD population [5]. Significant benefits have also been observed in patients requiring adjunctive treatment with mineralocorticoid receptor antagonists (MRAs) - an established treatment option for patients with persistent proteinuria, an important surrogate marker of ESRD [6]. However, despite their benefits, these drugs can compound the risk for hyperkalaemia among CKD patients [7–10]. Hyperkalaemia is often a major barrier for the optimal use of RAASi and/or MRAs in an already high-risk CKD population contributing to significant discrepancies between guideline recommendations and real-world practice in the use of RAASi and/or MRAs [8–11].

Hyperkalaemia is a clinically important electrolyte abnormality defined as a serum/plasma potassium level above the normal physiological range: 3.5–5.0 mmol/L (3.5–5.0 mEq/L) [12, 13]. Thresholds such as > 5.5, > 6.0 or > 7.0 mmol/L (mEq/L) are used to indicate severity [14]. As renal secretion is the main route of potassium elimination, patients with renal and metabolic comorbidities such as those with CKD or diabetes are at increased risk of hyperkalaemia. Whilst mild hyperkalaemia is usually asymptomatic, high levels of potassium are of concern to healthcare providers treating patients with CKD as it can induce electrophysiological disturbances that may cause life-threatening cardiac arrhythmias as well as muscle weakness or paralysis [15–18]. In the CKD population, hyperkalaemia is also associated with increased risk of hospitalisation, cardiac-related morbidity, and mortality [19–27].

There is currently a paucity of reliable treatment options for long-term serum potassium management and it has been suggested that best practise is to normalise serum potassium by down-titrating or discontinuing RAASi and/or MRA therapy in patients with cardiorenal disorders [28], despite evidence to suggest that submaximal dosing and discontinuation of RAASi are associated with increased risks of adverse outcomes in these patients [7, 29–31]. Furthermore, withholding RAASi treatment may lead to incremental healthcare costs associated with poor outcomes, for example, earlier onset of ESRD, hospitalizations due to cardiovascular causes, and cardiovascular mortality. Novel strategies that safely and effectively manage serum potassium levels, whilst allowing the optimal delivery of established renoprotective therapies, may therefore have significant potential to improve long-term outcomes in the CKD population.

With upcoming advances in pharmacological management of potassium levels for patients with cardiorenal syndromes, there is an increasing need to quantify the long-term health economic burden of hyperkalaemia in CKD, the consequences of suboptimal RAASi therapy, and the associated value of maintaining normokalaemia in this population. In the absence of long-term epidemiological evidence to describe hyperkalaemia in CKD, computer simulation modelling represents a valuable tool to simulate disease progression over a lifetime horizon, predict long-term outcomes, and inform clinical and reimbursement decision-making. Even though models characterising the natural history of CKD and predicting health economic outcomes associated with progression towards ESRD have been reported previously [32–37], none have modelled the inter-relationships between CKD progression, serum potassium levels, RAASi use, and the risk of adverse outcomes. The present study aimed to develop a novel model of CKD progression that incorporates serum potassium and RAASi use as variables to inform long-term health economic outcomes, and to quantify the value of maintaining normokalaemia and optimising RAASi therapy in advanced CKD patients at risk of hyperkalaemia.

## Methods

### Model development

A model was designed to characterise the natural progression of CKD patients (stage 3a onwards) and predict long-term health economic outcomes, which were associated with serum potassium levels and/or RAASi use. The model structure and inputs were informed by a targeted review of contemporary literature, UK clinical practice and National Institute for Health and Care Excellence (NICE) guidance where available. An advisory panel of four clinical experts, who were selected for their experiences in nephrology, cardiology, diabetes and general medicine, guided model conceptualisation and established the face validity of the model outputs.

### Model structure

**Fig. 1:**
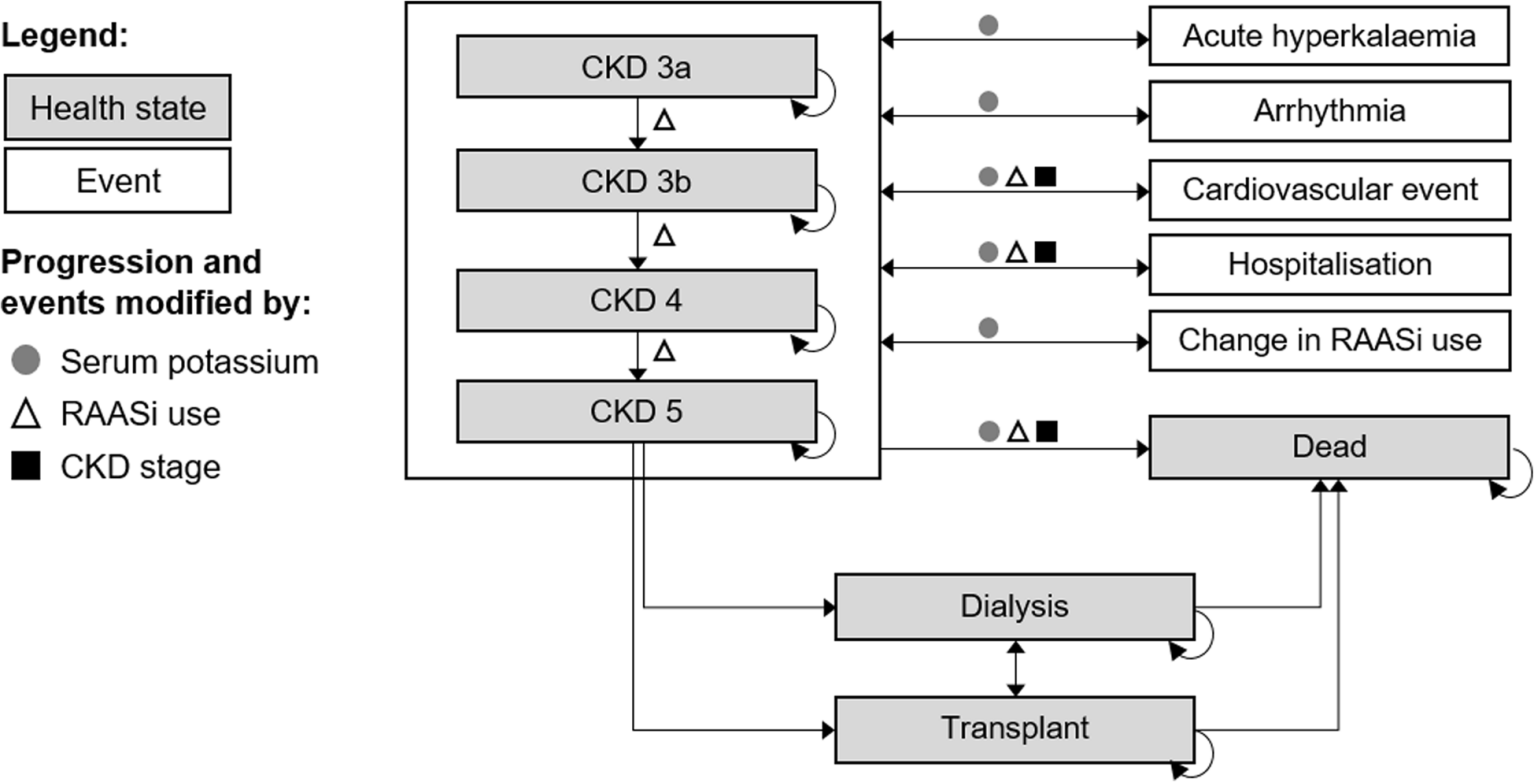
Flow diagram summarising CKD health states (shaded) and events (unshaded) applied in the model. Disease progression and the incidence of modelled events may be modified according to serum potassium level (grey circles), RAASi usage (white triangles) and/or CKD stage (black squares). CKD: chronic kidney disease; RAASi: renin-angiotensin-aldosterone system inhibitor

A patient-level simulation was developed in Microsoft Excel to capture the complex and chronic nature of CKD over a lifetime horizon (using a monthly cycle). Natural progression of CKD was modelled via a gradual decline in eGFR, where RAASi use impacted the rate of decline [4]. Similar to health states in previous CKD models [32–34], simulated patients progressed sequentially through CKD stages until ESRD onset (CKD stage 5; eGFR < 15 mL/min/1.73m^2^), the initiation of renal replacement therapy (RRT) (RRT; eGFR < 8.6 mL/min/1.73m^2^ [38]) and death from disease-specific or general causes (Fig. 1).

Whilst not influential to our illustrative analysis, the model can capture fluctuations in serum potassium commonly observed in CKD patients (further details in Additional file 1), acute hyperkalaemia events when predicted serum potassium levels exceed a specified threshold (e.g. > 6.5 mEq/L) and changes in RAASi use (discontinuation, down-titration or up-titration) according to serum potassium levels [11, 29].

Clinical events, and the effect of serum potassium levels and/or RAASi use on event risks, were modelled prior to RRT according to published relationships (Table 1). Baseline probabilities of hospitalisation, CV events and mortality were sourced by CKD stage [39] and the likelihood of arrhythmia among CKD patients was assumed equivalent to the heart failure population [40]. Associations between serum potassium and arrhythmia, CV events, hospitalisation and mortality were modelled using incidence rate ratios (IRRs) [11], while the impact of RAASi use on mortality and CV event incidence was modelled using odds ratios [5]. Background all-cause mortality was applied if it exceeded CKD-specific probabilities (adjusted for serum potassium level and/or RAASi use) according to gender-specific life tables [41].

**Table 1:** Model inputs to inform cardiovascular, hospitalisation and mortality event rate predictions in CKD patients

	CV event	Hospitalisation				Mortality	Source
Baseline annual event rate by CKD stage; mean (SE)^a^							
CKD 1–2	0.0211 (0.0012)	0.1354 (0.0045)				0.0076 (0.0002)	Go et al. [39]^c^
CKD 3a	0.0365 (0.0012)	0.1722 (0.0045)				0.0108 (0.0004)	
CKD 3b	0.1129 (0.0012)	0.4526 (0.0067)				0.0476 (0.0007)	
CKD 4	0.218 (0.0024)	0.8675 (0.0090)				0.1136 (0.0018)	
CKD 5 (ESRD)	0.366 (0.0048)	1.4461 (0.0090)				0.1414 (0.0031)	
Odds ratio by RAASi use; mean (SE)^b^							
Any versus none	0.820 (0.054)	1 (0)*				0.870 (0.069)	Xie et al.[5]^d^
Incidence rate ratio by serum potassium subgroup; mean (SE)^b^							
eGFR (mL/min/1.73m^2^):	Any	< 30	30–40	40–50	50–60	Any	Luo et al.[11]^e^
K^+^ < 3.5 mEq/L	1.89 (0.09)	1.93 (0.45)	1.77 (0.34)	2.24 (0.38)	2.06 (0.27)	3.05 (0.29)	
K^+^ 3.5–3.9 mEq/L	1.27 (0.04)	1.65 (0.22)	1.35 (0.16)	1.23 (0.13)	1.13 (0.08)	1.49 (0.08)	
K^+^ 4.0–4.4 mEq/L	1.04 (0.01)	0.93 (0.09)	0.99 (0.08)	1.08 (0.07)	1.01 (0.05)	1.06 (0.04)	
K^+^ 4.5–4.9 mEq/L	1 (0)	1 (0)	1 (0)	1 (0)	1 (0)	1 (0)	
K^+^ 5.0–5.4 mEq/L	1.01 (0.02)	1 (0.10)	0.96 (0.09)	1.07 (0.09)	1 (0.07)	1.14 (0.06)	
K^+^ 5.5–5.9 mEq/L	1.12 (0.04)	1.34 (0.18)	1.07 (0.14)	1.23 (0.18)	0.81 (0.11)	1.6 (0.13)	
K^+^ ≥ 6.0 mEq/L	1.88 (0.12)	3.65 (0.58)	1.82 (0.44)	1.91 (0.56)	1.07 (0.30)	3.31 (0.46)	

*CKD* chronic kidney disease, *CV* cardiovascular, *eGFR* estimated glomerular filtration rate, *ESRD* end stage renal disease, *RAASi* renin-angiotensin-aldosterone system inhibitor, *SE* standard error

^a^SE estimated from digitised plots showing 95% confidence intervals.

^b^SE estimated from 95% confidence intervals.

^c^Cardiovascular event defined in Go et al. [39] as: hospitalisation for coronary heart disease, heart failure, ischaemic stroke, and peripheral arterial disease.

^d^Cardiovascular event defined in Xie et al. [5] as: composite of fatal or nonfatal myocardial infarction, stroke, heart failure, cardiovascular death; or comparable definitions used by individual authors in studies included in the network meta-analysis.

^e^Luo et al. [11] reported incidence rate ratios (IRRs) for a major adverse cardiovascular event (MACE); these values were applied to the risk of both arrhythmia and cardiovascular events.

*Null value; no evidence identified

This study aimed to estimate the value of maintaining normokalaemia irrespective of the strategy used to achieve this target, therefore costs and utilities related to pharmacological serum potassium management were not considered. For all other costs and benefits applied in the illustrative analyses, a UK healthcare payer perspective was adopted. Healthcare resource costs were obtained from published sources [1, 40, 42–46] and inflated to 2014–15 GBP [47]. Health-related quality of life was estimated via the multiplicative application of published health state and event utilities [48–55] to an age-dependent baseline value [56].

A summary of the methods used to model CKD progression and events is provided in Additional file 2: Table S1, an illustration of modelled cumulative event incidence for different patient characteristics in Additional file 3: Figure S1, and the inputs applied to modelled health states and events in Additional file 2: Table S2.

### Model validation

To assess the validity of the model’s predictions, the modelled incidence of death and major adverse cardiovascular events (MACE) were used to derive modelled IRRs as a function of serum potassium level, which were compared to IRRs published by Luo et al. [11] (internal validation) and unadjusted IRRs derived from a retrospective, observational cohort study of CKD patients listed on the UK Clinical Practice Research Datalink (CPRD) [57, 58] (external validation).

### Model application

The model was used to estimate the consequences of discontinuing RAASi therapy to maintain normal potassium levels in advanced CKD patients in terms of lifetime healthcare costs, life-expectancy and quality-adjusted life years (QALYs). Analysis was conducted for a cohort of CKD stage 3a patients (eGFR 52.5 mL/min/1.73 m2), who were aged 60 years at baseline. Serum potassium was maintained at 4.5 mEq/L for all patients. Though the treatment arm represented a cohort of patients who received optimal serum potassium management to enable the continuation of RAASi therapy, the cost of such strategies (pharmacological and/or monitoring) was not included. All other costs and benefits were discounted at 3.5% per annum [59].

The health economic value of maintaining normokalaemia and optimising RAASi therapy was summarised in terms of incremental net monetary benefit (NMB) which was derived using willingness-to-pay (WTP) thresholds of £20,000–30,000 per QALY gained, in line with UK assessments of cost-effectiveness. In this analysis, incremental NMB represents the amount of money that could be spent on strategies to maintain normokalaemia that would be deemed good value for money.

### Sensitivity and scenario analyses

The sensitivity of model predictions to changes in the following variables were assessed: time horizon (5, 10, 15 or 20 years); age (60 ± 20 years); CKD stage 3a (eGFR 52.5 mL/min/1.73m^2^), 3b (eGFR 37.5 mL/min/1.73m^2^) or 4 (eGFR 22.5 mL/min/1.73m^2^); male or female; RAASi efficacy for CKD progression (0–100% of base case), events (0–100% of base case), or both (base case ±20%); transplant probability, RRT mortality rate, event costs, event disutility, CKD 3–5 costs, CKD 3–5 utility, RRT costs and RRT utility (all base case ±20%).

Additional scenario analyses were conducted to investigate the impact of RAASi dosing on predicted outcomes. In the base case, patients continuing RAASi therapy were assumed to receive optimal dosing and consequently achieve the full benefit of RAASi observed in intention-to-treat trial populations [4, 5]. Though suboptimal dosing is common in clinical practice, there is a paucity of published data to describe the efficacy associated with such dosing; therefore, model parameters describing the cost and efficacy of RAASi treatment (related to CKD progression, CV and mortality risk) were scaled from 0 to 100% of base case values.

## Results

### Model validation

**Fig. 2:**
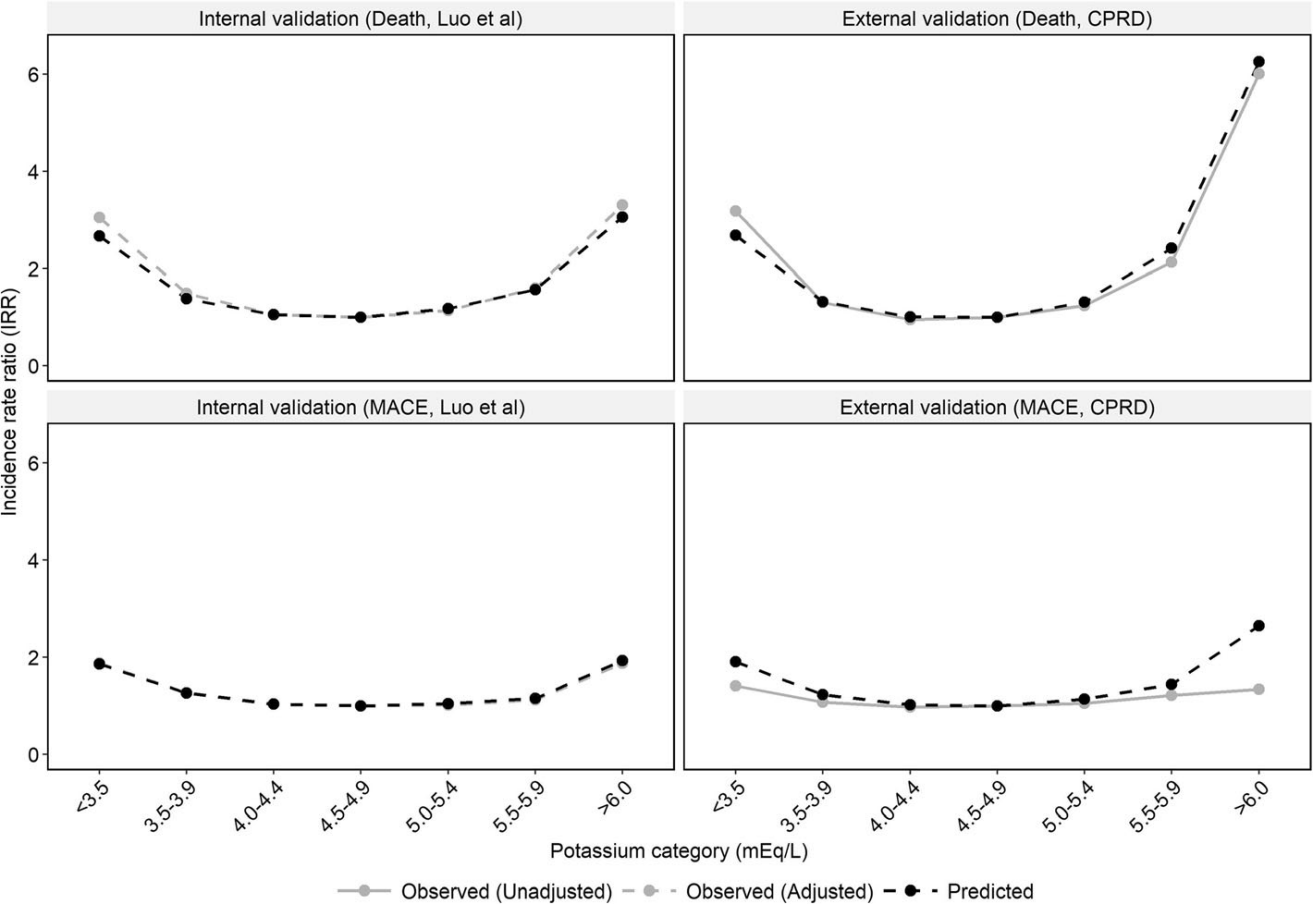
Comparison of predicted IRRs for death and MACE, as a function of serum potassium level, against IRRs from other studies. Grey lines indicate IRRs from observational studies; black lines indicate predicted IRRs. IRRs reported by Luo et al. were adjusted for selected patient attributes. *CPRD* Clinical Practice Research Datalink, *IRR* incidence rate ratio, *MACE* major adverse cardiovascular event

The model reproduced the associations between serum potassium and incident clinical outcomes from observational studies (Fig. 2). Internal validation exercises found that predicted IRRs for death and MACE were consistent with adjusted IRRs reported by Luo et al. [11] (R^2^: 0.995 and 0.995, respectively). External validation exercises against CPRD data showed that the model had a high predictive capability when estimating associations between serum potassium and mortality (R^2^: 0.961), while differences between predicted and observed IRRs for MACE may be due to the potential under-reporting of fatal MACE in the CPRD (R^2^: 0.743).

### Model application

In modelled CKD patients who received optimal serum potassium management to enable ongoing RAASi therapy, predicted mean life expectancy was extended by 2.36 years when compared with patients who discontinued RAASi to maintain normokalaemia (Table 2). Ongoing RAASi treatment was associated with delayed CKD progression; average time spent in CKD stage 3a, 3b and 4 was longer in the treatment arm than in the control arm (by 1.05, 1.69 and 0.86 years, respectively), while fewer patients receiving RAASi were predicted to reach ESRD within their lifetime (40% versus 49% in the control arm). Of those who progressed to ESRD, the estimated time to ESRD onset was delayed by 5.4 years in patients who received optimal serum potassium management and continued RAASi therapy (16.2 years versus 10.8 years without RAASi treatment). Similarly, the estimated time to RRT initiation was delayed by 6.2 years in the treatment arm, and the percentage of patients predicted to reach RRT over a lifetime was reduced by 9%.

**Table 2:** Predicted value of optimal serum potassium management to enable ongoing RAASi therapy in CKD patients

Discounting	Outcome	Treatment arm (ongoing RAASi)	Control arm (no RAASi)	Incremental benefit	Incremental NMB	
					WTP at £20,000 per QALY	WTP at £30,000 per QALY
Undiscounted	Cost	£108,685	£108,720	-£35	£28,570	£42,837
	QALY	8.60	7.17	1.43		
	LY	15.12	12.75	2.36		
Discounted	Cost	£72,067	£75,202	-£3135	£23,446	£33,601
	QALY	6.68	5.67	1.02		
	LY	11.32	9.77	1.56		

*CKD* chronic kidney disease, *ESRD* end stage renal disease, *LY* life-year, *NMB* net monetary benefit, *QALY* quality-adjusted life-year, *RAASi* renin-angiotensin-aldosterone system inhibitor, *WTP* willingness-to-pay threshold

Simulated patients were aged 60 years at baseline, with CKD stage 3a (eGFR 52.5 mL/min/1.73m^2^) and serum potassium of 4.5 mEq/L; analyses were conducted independent of costs and utilities related to pharmacological potassium management

**Fig. 3:**
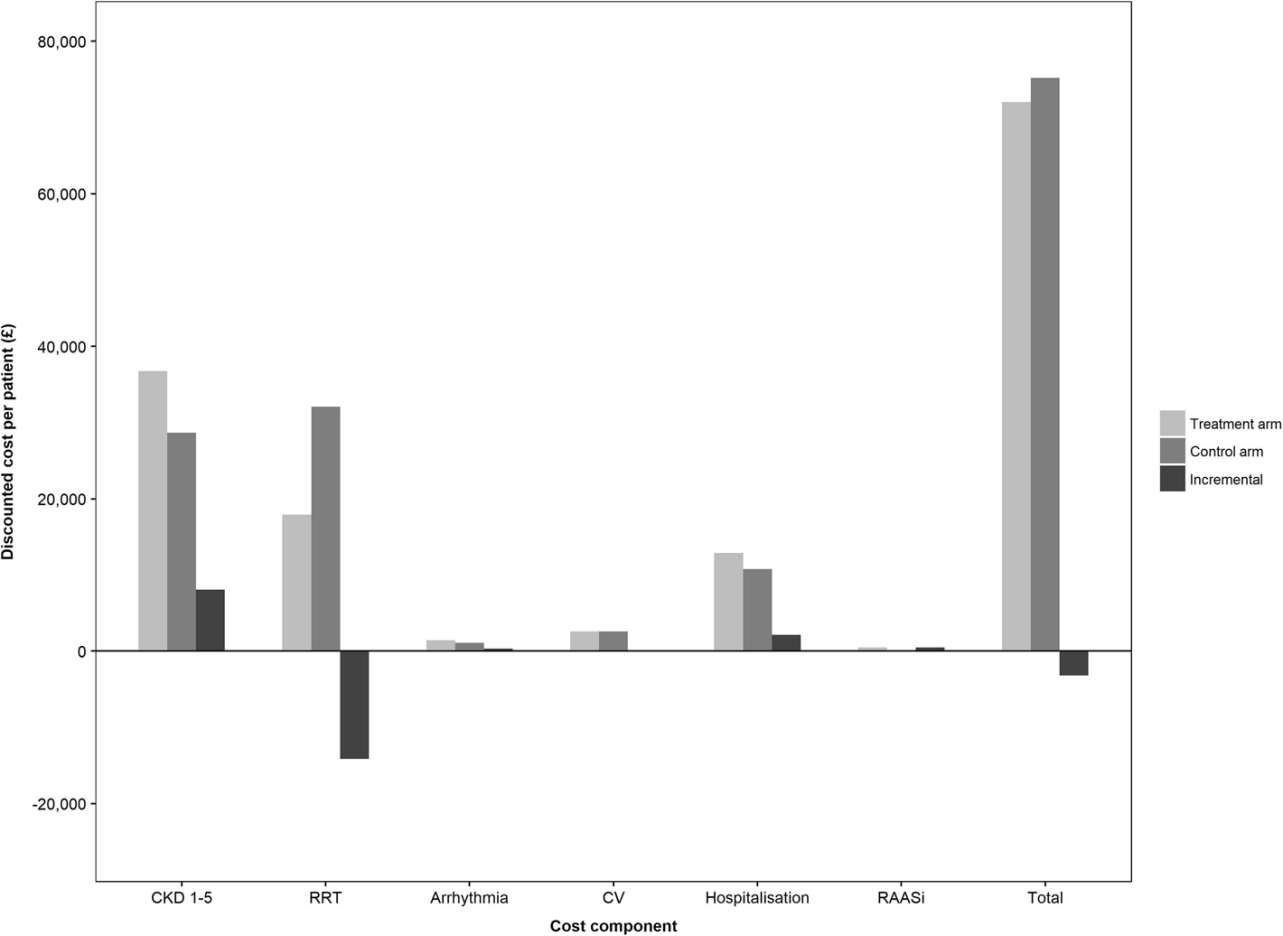
Discounted cost breakdown for modelled CKD patients receiving optimal serum potassium management to enable ongoing RAASi therapy (treatment arm), compared against patients not receiving RAASi to maintain normokalaemia (control arm). *CKD* chronic kidney disease, *CV* cardiovascular event, *RAASi* renin-angiotensin-aldosterone system inhibitor, *RRT* renal replacement therapy

Over a lifetime horizon, optimisation of both serum potassium levels and RAASi therapy was associated with per-patient cost savings of £3135 (£35 undiscounted), and QALY gains of 1.02 (1.43 undiscounted) (Table 2). As shown in Fig. 3, predicted cost savings in this cohort were largely driven by the avoidance of RRT (£14,143 per patient), while additional per-patient costs related to CKD management (+£8091), arrhythmia (+£327) and hospitalisation (+£2129) were attributed to improved survival and extended exposure to such risks. Overall, predicted cost savings and QALY gains corresponded to incremental NMB of £23,446 and £33,601, which represent the amount that could be spent on cost-effective strategies to maintain normokalaemia, at WTP thresholds of £20,000 and £30,000 per QALY gained, repectively (Table 2).

### Sensitivity and scenario analysis

**Fig. 4:**
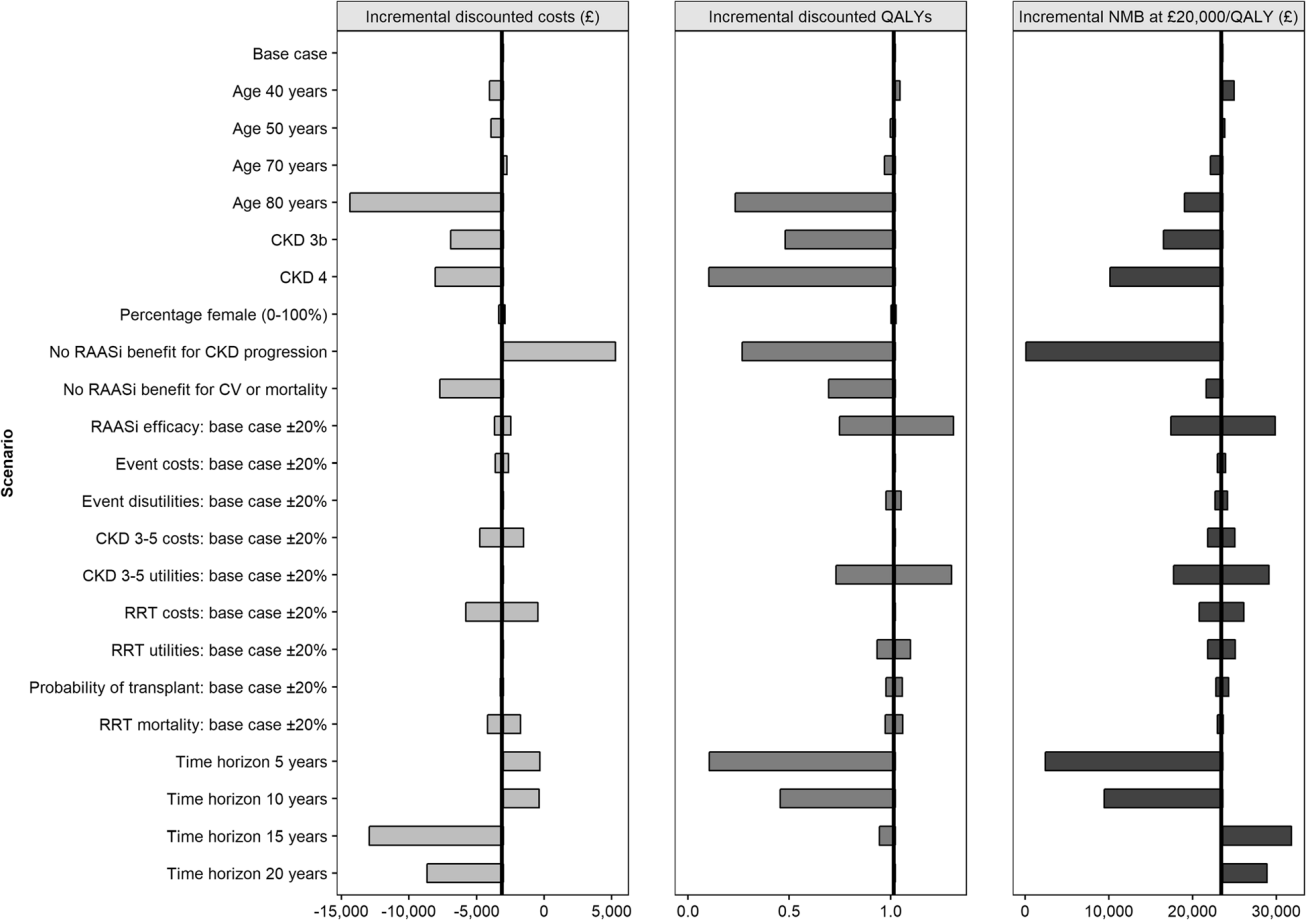
Impact of model inputs on incremental discounted costs, QALYs and NMB (WTP at £20,000 per QALY gained). *CKD* chronic kidney disease, *CV* cardiovascular event, *NMB* net monetary benefit, *QALY* quality-adjusted life-year, *RAAS*i renin-angiotensin-aldosterone system inhibitor, *RRT* renal replacement therapy, *WTP* willingness-to-pay threshold

Figure 4 summarises the results of deterministic sensitivity analyses. The predicted value of maintaining normokalaemia and RAASi therapy was greatest for CKD patients aged 40 years at baseline (£24,979 incremental NMB at £20,000 per QALY), and progressively decreased with increasing cohort age (£19,037 at 80 years of age); this was largely attributed to lower QALY gains predicted for older patients. Similarly, lower predicted QALY gains led to lower incremental NMB in patients with more advanced CKD at baseline (£23,446, £16,532 and £10,139 in CKD stage 3a, 3b and 4, respectively). As the modelled time horizon increased from 5 to 20 years, progressively greater QALY gains were captured; however, the most favourable cost savings (−£12,942) and incremental NMB (£31,831) were estimated at 15 years, driven by the timing of ESRD and initiation of RRT. Predicted incremental costs, QALYs and NMB were also sensitive to the modelled efficacy of RAASi therapy, particularly in relation to CKD progression. When the cost and efficacy of RAASi were scaled from 0 to 100% of base case values, discounted QALYs, life-years and NMB improved with increasing RAASi efficacy, while the incidence of ESRD and RRT, and associated costs, decreased with improved RAASi efficacy (Additional file 3: Figure S2).

**Fig. 5:**
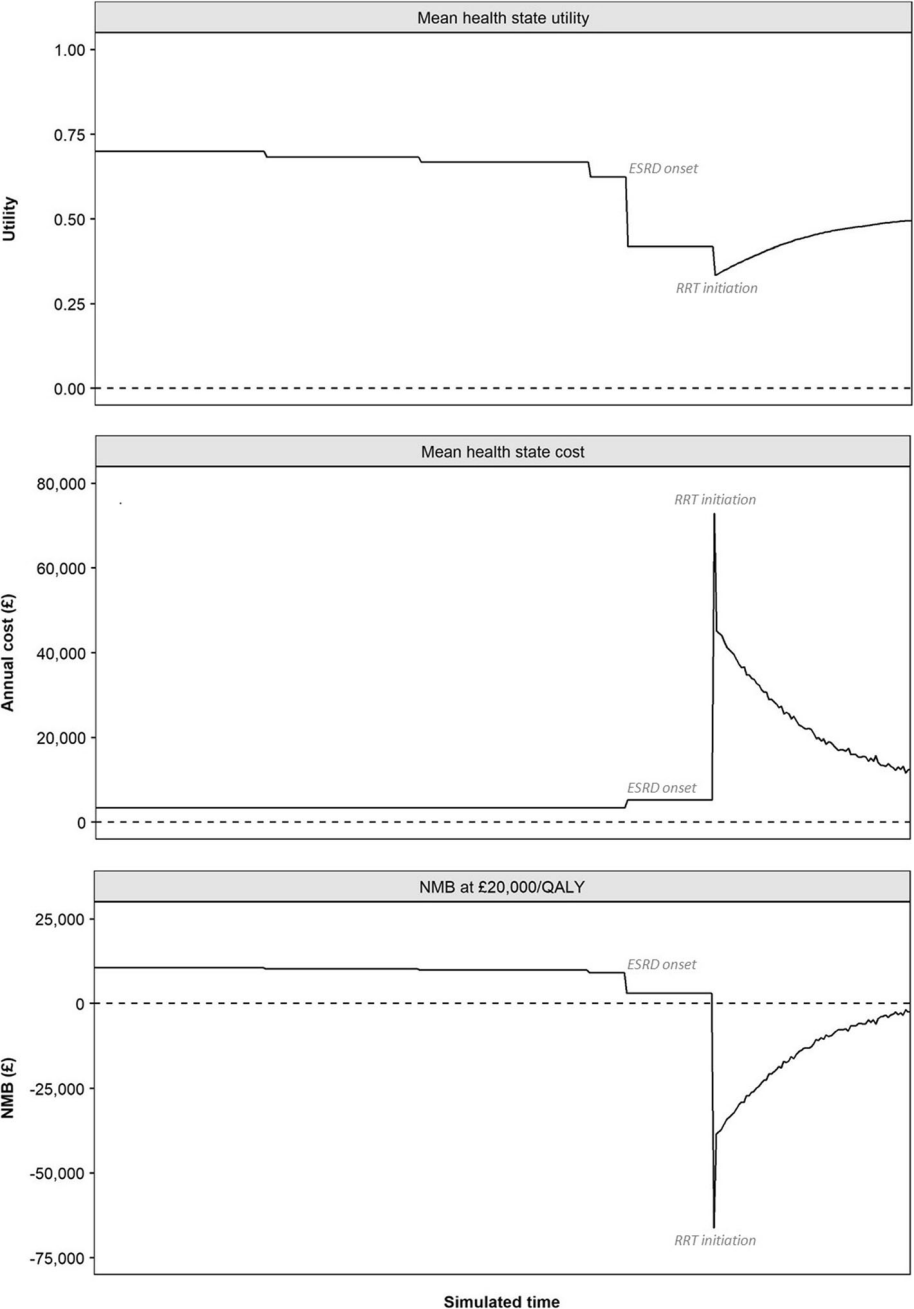
Evolution of mean health state utility, undiscounted health state costs and associated absolute NMB (WTP at £20,000 per QALY gained) over 20 years in surviving CKD patients. *CKD* chronic kidney disease, *ESRD* end stage renal disease, *NMB* net monetary benefit, *QALY* quality-adjusted life-year, *RRT* renal replacement therapy, *WTP* willingness-to-pay threshold

Sensitivity analyses suggested that the timing and cost of ESRD, and subsequent RRT, were significant drivers of health economic outcomes. Figure 5 illustrates the evolution of mean health state utility (age-adjusted), undiscounted health state costs, and associated absolute NMB, which represents the undiscounted monetary value of one additional year in each health state. Among surviving CKD patients in the treatment arm, estimated NMB was £3057–£10,585 in the 18.8 years prior to RRT initiation, resulting from comparatively high mean health state utility (0.42–0.70) and low health state costs (£3404–£5311) associated with CKD stages 3a–5 (pre-dialysis). In contrast, the initiation of RRT was associated with lower health state utility and significantly increased health state costs, resulting in negative NMB (−£66,175 NMB at £20,000 per QALY). Estimated NMB among surviving RRT patients was observed to increase over time, as fewer patients received dialysis due to increased transplantation and death. Nevertheless, negative NMB estimated following the initiation of RRT served to significantly offset the value associated with increased survival prior to ESRD.

## Discussion

Hyperkalaemia has been associated with increased risks of mortality and morbidity and with the dose down-titration or discontinuation of RAASi among CKD patients, which in turn is associated with worsening clinical outcomes. To our knowledge, this is the first study to describe the development of a natural history model for CKD that utilised serum potassium and RAASi use as variables to inform long-term health economic outcomes. The model represents a valuable resource to assess the health economic burden of hyperkalaemia in CKD, quantify the value of serum potassium management, and inform future decision making. Findings from applications of the model are consistent with those recently reported by Bakhai et al., which demonstrated the value of potassium management to avoid hyperkalaemia, enable RAASi therapy and improve long-term health economic outcomes in patients with heart failure [60].

The accuracy and generalisability of the model predictions are limited by the inputs used to inform its development and several assumptions were necessary due to a paucity of published literature. For this reason, the model does not capture associations between RAASi use and hospitalisation or arrhythmia; and our base case analyses reflect a scenario in which patients received RAASi doses recommended in treatment guidelines for heart failure [42]. This study focussed on RAASi treatment due to its use as first line therapy, and the use of MRAs was not included in analyses. Consequently, the increased risk of hyperkalaemia associated with adjunctive MRA treatment and the value of maintaining normokalaemia to enable appropriate prescription of MRAs has not been captured.

The true value of optimal serum potassium management in CKD patients may be underestimated by current model predictions, as other clinical events and downstream benefits (such as the avoidance of implantable cardiac devices through reduced arrhythmia incidence) were not considered due to lack of published evidence but may be modelled if such data become available. Studies used to inform the incidence of hospitalisation did not stratify hospitalisations by cause [11, 39]; consequently, total admissions may be overestimated due to the inclusion of hospitalisations and events that may require hospitalisation (e.g. cardiovascular events) without available evidence to adjust for any potential double counting. Furthermore, an average linear decline was applied to model progression of eGFR. Factors such as age, sex, hypertension, acute kidney injury, diabetes or proteinuria may influence rates of renal function decline and associated non-linear trajectories of eGFR are an area for further research and expansion of the model scope. Presence or absence of proteinuria was not modelled, however is also relevant considering guideline recommendations for prescription of RAASi therapy in CKD with urine albumin excretion > 30 mg/24 h (diabetics) and > 300 mg/24 h (non-diabetics) [2].

The presented validation exercises were limited to a comparison of modelled IRRs against observed values, due to a current paucity of literature available to validate model predictions of absolute outcomes. However, as new data describing the relationships between serum potassium, clinical events and long-term outcomes in CKD become available, ongoing model refinement and validation will ensure the continuing accuracy of its predictions. Finally, our analyses were conducted independent of pharmacological potassium management and/or monitoring costs, making the analysis relevant to any intervention, noting that interventions aimed to maintain normokalaemia in CKD are likely to incur additional expenditure. Extending the model to incorporate treatment costs and effects will allow the value of specific serum potassium management strategies to be quantified in future health economic analyses.

To illustrate the functionality of the model, we showed that patients who maintained normokalaemia and received ongoing RAASi therapy were predicted to benefit from delayed CKD progression, ESRD onset and RRT initiation, improved life expectancy, overall cost savings, and QALY gains, compared to those who discontinued RAASi to achieve equivalent serum potassium levels. However, as RAASi treatment is a known cause of hyperkalaemia careful monitoring for hyperkalaemia may be required to ensure a timely intervention should potassium levels rise. This requirement to monitor potassium may add to the burden of healthcare providers and patients and could hinder RAASi use in clinical practice.

Currently available approaches to restore potassium homeostasis in patients treated with RAASi are limited to dietary potassium restriction, correction of metabolic acidosis with use of bicarbonate solutions and increasing doses of loop diuretics to enhance renal potassium secretion, whilst recent advances include potassium-exchanging resins to bind potassium in the gut [61–63]. Potassium-binding agents can improve hyperkalaemia management whilst providing the opportunity for patients with CKD to benefit from the full cardio- and renoprotective effects of RAASi [64]. The model developed in this study has the potential to inform broad decision making on interventions for hyperkalaemia, as cost is an increasingly important consideration. To allow generalisability across a range of clinical scenarios, the presented model application did not focus on a single intervention and estimates of NMB from this study may be compared against the cost of any intervention to maintain normokalaemia and enable optimal RAASi therapy.

Sensitivity analyses demonstrated that greater value was expected among simulated patients of younger age and/or earlier CKD stage. Scenario analyses highlighted the detrimental impact of suboptimal RAASi dosing on clinical and economic outcomes. Though maintenance of lower doses of RAASi therapy are expected to provide some benefit, reduced efficacy relative to optimal dosing was associated with modelled increases in incidence of ESRD and RRT, and associated costs, and decreases in discounted QALYs, life-years and NMB. The model has flexibility for future applications investigating other scenario analyses.

Prolonged survival among the CKD population may increase the number of patients reaching ESRD and requiring RRT; a modality of care that may be considered not cost-effective due to its high costs and poor quality of life. Data arising from this study, whereby negative NMB associated with RRT partially offset the benefit of treatment prior to ESRD, subsequently illustrate the potential for RRT to confound the cost-effectiveness of health technologies for CKD. This observation is consistent with the notion that the inclusion of RRT costs may distort the results of economic analyses, and thus deny the availability of efficacious treatments that extend the life of CKD patients but do not directly impact the requirement for dialysis [65]. While others present a case for excluding dialysis costs in such evaluations [65], we alternatively propose that health economic outcomes in CKD may be more accurately described when partitioned according to ESRD status.

## Conclusions

The model developed in this study is the first to our knowledge to characterise the natural history of CKD as a function of serum potassium levels and adherence to RAASi therapy, and highlights the current health economic consequences of underutilising RAASi to avoid hyperkalaemia. The optimisation of both serum potassium and RAASi therapy was associated with delayed CKD progression and initiation of RRT, improved quality of life and survival, and overall cost savings. Analyses were not intended to quantify the cost-effectiveness nor support the adoption of a specific treatment approach, but rather support the notion that maintaining normokalaemia (using guideline-recommended treatments, based on best available evidence) is a valuable strategy to enable optimal RAASi therapy and improve long-term health economic outcomes in CKD patients.

## Additional files

Additional file 1: Supplementary Methods Mixed-effects model for serum potassium profiles. (DOCX 15 kb)

Additional file 2: **Table S1.** Summary of methods employed to model disease progression and events in CKD patients. **Table S2.** Inputs applied to modelled health states and events. (DOCX 37 kb)

Additional file 3: **Figure S1.** Model-estimated cumulative events over five years, according to CKD stage (a–c), RAASi use (d–f) and incidence of hyperkalaemia (g–i). **Figure S2.** Impact of RAASi cost and efficacy (0–100% of base case values) on discounted lifetime per-patient costs, QALYs, life years and NMB at conventional WTP thresholds. (DOCX 453 kb)

## Abbreviations

ACE: Angiotensin converting enzyme
ARB: Angiotensin receptor blockers
CKD: Chronic kidney disease
CPRD: Clinical Practice Research Datalink
CV: Cardiovascular
eGFR: Estimated glomerular filtration rate
ESC: European Society of Cardiology
ESRD: End stage renal disease
IRR: Incidence rate ratios
MACE: Major adverse cardiovascular events
MIMS: Monthly Index of Medical Specialities
MRA: Mineralocorticoid receptor antagonist
NICE: National Institute for Health and Care Excellence
NMB: Net monetary benefit
QALY: Quality-adjusted life year
RAASi: Renin-angiotensin-aldosterone system inhibitor
RRT: Renal replacement therapy
WPT: Willingness-to-pay
