# Supplementary material for: The value of maintaining normokalaemia and enabling RAASi therapy in chronic kidney disease
Source: BMC Nephrol. 2019 Jan 31;20:31. doi: 10.1186/s12882-019-1228-y (PMC6357372; doi:10.1186/s12882-019-1228-y)
Supplement: Supplementary file 1 — Supplementary Methods Mixed-effects model for serum potassium profiles. (DOCX 15 kb) [file 12882_2019_1228_MOESM1_ESM.docx]

## Supplementary Methods

## Mixed-effects model for serum potassium profiles

To quantify the health economic value of optimal serum potassium (K^+^) management, the present study compared CKD patients with sustained normokalaemia (K^+^ 4.5 mEq/L) and ongoing RAASi therapy, against those who discontinued RAASi to maintain normokalaemia. However, to simulate more complex, fluctuating serum K^+^ profiles in patients at risk of hyperkalaemia, time-dependent serum K+ trajectories may be modelled at the patient-level using mixed-effects regression models. Fixed effects model parameters capture overall potassium trends at the cohort-level, while random effects terms allow individual patients to exhibit a unique, fluctuating potassium profile that may deviate from the cohort average.

Mixed-effects regression models are of the form:

$$K_{ij}^{+}=\left( \alpha+\upsilon_{i} \right)+\sum_{k} \left( \beta_{k}+\gamma_{ik} \right)\times{Time}_{ijk}+\varepsilon_{ij}$$

$$\upsilon_{i} \sim N\left( 0,\sigma_{\upsilon_{i}}^{2} \right) ; \gamma_{ik} \sim N\left( 0,\sigma_{\gamma_{ik}}^{2} \right) for each k ; \varepsilon_{ij}\sim N(0,\sigma_{\varepsilon_{ij}}^{2})$$

Where $K_{ij}^{+}$ represents a K^+^ measurement for patient $i$ made at time $j$; ${Time}_{ijk}$ is a time index of specification $k$, representing patient $i$‘s time at measurement occasion $j$; $\alpha$ and $\beta_{k}$ are fixed intercept and slope coefficients respectively, representing population-averaged baseline K^+^ concentration and the association between K^+^ and time; $\upsilon_{i}$ and $\gamma_{ik}$ are random intercept and slope terms respectively, representing patient-specific effects for patient $i$ (allowing levels and slopes of K^+^ trajectories to vary by patient); and $\varepsilon_{ij}$ is a random error term for patient $i$ at measurement occasion $j$, capturing all sources of variation in K^+^ not explained by the model.

The measurement-level (level 1) random error term $\varepsilon_{ij}$ is assumed to be normally distributed with mean 0 and constant variance $\sigma_{\varepsilon_{ij}}^{2}$. Like $\varepsilon_{ij}$, $\upsilon_{i}$ and $\gamma_{ik}$ are error terms with constant patient-level (level 2) variances $\sigma_{\upsilon_{i}}^{2}$ and $\sigma_{\gamma_{ik}}^{2}$ respectively; it is these patient-level variances that must be estimated in the model to provide estimates of the random effects, in contrast to $\alpha$ and $\beta_{k}$ which are global coefficients to be estimated to provide estimates of the fixed effects. The total variance in K^+^ about the population-averaged time trend is equal to the sum of the individual level 1 and level 2 variance components, $\sigma^{2}=\sigma_{\upsilon_{i}}^{2}+\sum_{k} \sigma_{\gamma_{ik}}^{2}+\sigma_{\varepsilon_{ij}}^{2}$. All else being equal: larger values of $\sigma_{\upsilon_{i}}^{2}$ will increase the spread of patient-specific mean values about the global time trend; larger values of $\sigma_{\gamma_{ik}}^{2}$ will increase the spread of the slopes of patient-specific time trends; and larger values of $\sigma_{\varepsilon_{ij}}^{2}$ will increase the spread of measured K^+^ values about patient-specific time trends.
