# Supplementary material for: The value of maintaining normokalaemia and enabling RAASi therapy in chronic kidney disease
Source: BMC Nephrol. 2019 Jan 31;20:31. doi: 10.1186/s12882-019-1228-y (PMC6357372; doi:10.1186/s12882-019-1228-y)
Supplement: Supplementary file 2 — Table S1. Summary of methods employed to model disease progression and events in CKD patients. Table S2. Inputs applied to modelled health states and events. (DOCX 37 kb) [file 12882_2019_1228_MOESM2_ESM.docx]

Table S1 Summary of methods employed to model disease progression and events in CKD patients

| **Events** | **Baseline incidence rate** | **Modified by RAASi use?** | | **Modified by K^+^ levels?** | |
| --- | --- | --- | --- | --- | --- |
| Progression (eGFR) | Annual rate of decline; Evans et al. [1] | ✓ | Any versus none; Evans et al. [1] | 🗶 |  |
| Acute hyperkalaemia | Incidence of K^+^ above threshold | 🗶 |  | ✓ | By definition |
| RAASi discontinuation | By RAASi dose level; Epstein et al. [2] | ✓ | By definition | ✓ | By K^+^ category; Epstein et al. [2] |
| RAASi down-titration | By RAASi dose level; Epstein et al. [2] | ✓ | By definition | ✓ | By K^+^ category; Epstein et al. [2] |
| Arrhythmia | Monthly probability; Colquitt et al. [3] | 🗶 |  | ✓ | MACE IRR; Luo et al. [4] |
| Cardiovascular | By CKD stage; Go et al. [5] | ✓ | OR any versus none; Xie et al. [6] | ✓ | MACE IRR; Luo et al. [4] |
| Hospitalisation | By CKD stage; Go et al. [5] | ✓ | Appropriate data not identified | ✓ | Any hospitalisation IRR; Luo et al. [4] |
| Mortality^a^ | By CKD stage; Go et al. [5] | ✓ | OR any versus none; Xie et al. [6] | ✓ | Any mortality IRR; Luo et al. [4] |
| *CKD: chronic kidney disease; eGFR: estimated glomerular filtration rate; IRR: incidence rate ratio, K^+^: potassium; MACE: major adverse cardiovascular event; OR: odds ratio; RAASi: renin-angiotensin-aldosterone system inhibitor; ✓: functionality; 🗶: no functionality (due to paucity of identified data)*   1. The higher probability based on (A) comorbidity, RAASi use and K^+^ levels, or (B) life tables is applied throughout. | | | | | |

Table S2 Inputs applied to modelled health states and events

|  | **Value** | **Source** |
| --- | --- | --- |
| **RRT health state inputs** | | |
| eGFR threshold for RRT initiation (mL/min/1.73m^2^) | 8.600 | UK Renal Registry [7] |
| Annual probability of dialysis complications | 0.080 | UK Renal Registry [7]; NICE CG125 [8] |
| Probability of death following dialysis complications | 0.004 | UK Renal Registry [7]; NICE CG125 [8] |
| Maximum number of transplants per patient | 2 | Assumption |
| Annual probability of transplant | 0.244 | NHSBT [9]^d^ |
| Annual probability of dialysis death | 0.2 | UK Renal Registry [7]^e^ |
| Annual probability of graft failure | 0.025 | NHSBT [9] |
| Annual probability of transplant death | 0.020 | NHSBT [9] |
| **Health state utilities** | | |
| CKD 3a | 0.870 | Gorodetskaya et al. [10] |
| CKD 3b | 0.870 | Gorodetskaya et al. [10] |
| CKD 4 | 0.850 | Gorodetskaya et al. [10] |
| CKD 5 (ESRD; pre-RRT) | 0.570 | Lee et al. [11] |
| Dialysis | 0.452 | Lee et al. [11]^f^ |
| Transplant | 0.710 | Lee et al. [11] |
| **Event disutilities^a^** | | |
| Arrhythmia | -0.025 | Sullivan et al. [12] |
| CV event (year 1) | -0.321 | BHF [13]; Haacke et al. [14]; Holland et al. [15]; Lacey et al. [16]^g^ |
| CV event (year 2+) | -0.321 | BHF [13]; Haacke et al. [14]; Holland et al.[15]; Lacey et al. [16]^g^ |
| Hospitalisation | -0.024 | Göhler et al. [17] |
| Dialysis complications | -0.060 | NICE CG125 [8]; Sennfalt et al. [18] |
| **Health state costs (£)**^b^ | | |
| Annual cost of CKD 3a | 3,404.11 | NICE CG182 [19] |
| Annual cost of CKD 3b | 3,404.11 | NICE CG182 [19] |
| Annual cost of CKD 4 | 3,404.11 | NICE CG182 [19] |
| Annual cost of CKD 5 (ESRD; pre-RRT) | 5,311.08 | NICE CG182 [19] |
| Annual cost of RAASi therapy | 45.88 | ESC [20]; MIMS [21]^h^ |
| Annual cost of dialysis | 34,358.45 | Baboolal et al. [22] |
| Dialysis access cost | 2,051.25 | NHS reference costs [23]; weighted by dialysis access modality |
| One-off cost of dialysis complications | 3,877.24 | NICE CG125 B [8]; weighted by dialysis modality |
| One-off cost of transplant procedure^c^ | 13,965.66 | NHS reference costs [23]; weighted by donor type |
| One-off Organ Transplantation Service cost | 16,009.80 | Calculated^i^ |
| Annual cost of transplant maintenance | 6,653.38 | NICE CG125 [8] |
| **Event costs (£)^b^** | | |
| Arrhythmia | 1,453.32 | Colquitt et al. [3] |
| CV event | 2,336.91 | BHF [13]; NHS reference costs [23]^j^ |
| Hospitalisation | 2,444.80 | Colquitt et al. [3]; assumed equal to heart failure population |
| *BHF: British Heart Foundation; CKD: chronic kidney disease; CV: cardiovascular; ESC: European Society of Cardiology; ESRD: end-stage renal disease; MIMS: Monthly Index for Medical Specialities; NHS: National Health Service; NHSBT: National Health Service Blood and Transplant; NICE: National Institute for Health and Care Excellence; RAASi: renin-angiotensin-aldosterone inhibitor; RRT: renal replacement therapy*   1. Event disutility values (subtracted from 1) were applied multiplicatively to baseline values. 2. Costs were inflated to 2014–15 values using the Personal Social Services Research Unit (PSSRU) Hospital and Community Health Services (HCHS) pay and price inflation index [24]. 3. One-off cost of a renal transplant procedure was applied at the point of entry into the transplant health state; ongoing transplant maintenance costs were incurred thereafter. 4. Annual probability of transplant was based on the median waiting time reported for the UK [9], and was assumed to be consistent across age groups (<35; 35-44; 45-54; 55-64; 65+ years). 5. Annual probability of dialysis death among incident RRT patients aged ≥65 years. 6. Health state utility for dialysis reflects a weighted average, based on the proportion of patients receiving peritoneal dialysis and haemodialysis reported by Lee et al. [11]. 7. Disutility for CV events reflects a weighted average, based on the UK prevalence of stroke, heart failure and myocardial infarction reported by the British Heart Foundation [13], and corresponding utility values for each [14-16]. A constant disutility was assumed for the year of the CV event (year 1), and subsequent years thereafter (year 2+). 8. Annual cost of RAASi therapy reflects a weighted average, based on maximum daily doses recommended in European Society of Cardiology (ESC) guidelines for HF [20], and costs sourced from MIMS [21]. It was assumed that 90% of CKD patients in UK clinical practice receive angiotensin-converting enzyme inhibitors (assumed ramipril), 10% receive angiotensin II receptor blockers (assumed candesartan) and 50% receive mineralocorticoid receptor antagonists as additive therapy (assumed spironolactone). 9. Costs associated with organ donation and transplant activities (2011–12) were obtained by Freedom of Information request. The annual expenditure was divided by the number of transplants performed in 2011­–12 to derive a cost per transplant, then inflated to 2014–15 prices. 10. Disutility for CV events reflects a weighted average, based on the UK prevalence of stroke, heart failure and myocardial infarction reported by the British Heart Foundation [13]; and activity and unit costs for each sourced from NHS reference costs 2014–15 [23]. | | |

**References**

1. Evans M, Bain SC, Hogan S, Bilous RW. Irbesartan delays progression of nephropathy as measured by estimated glomerular filtration rate: post hoc analysis of the Irbesartan Diabetic Nephropathy Trial. Nephrol Dial Transplant. 2012;27(6):2255-63.

2. Epstein M, Reaven NL, Funk SE, et al. Evaluation of the treatment gap between clinical guidelines and the utilization of renin-angiotensin-aldosterone system inhibitors. Am J Manag Care. 2015;21(Suppl 11):s212-20.

3. Colquitt JL, Mendes D, Clegg AJ, et al. Implantable cardioverter defibrillators for the treatment of arrhythmias and cardiac resynchronisation therapy for the treatment of heart failure: systematic review and economic evaluation. Health Technol Assess. 2014;18(56)1-560.

4. Luo J, Brunelli SM, Jensen DE, Yang A. Association between serum potassium and outcomes in patients with reduced kidney function. Clin J Am Soc Nephrol. 2016;11(1):90-100.

5. Go AS, Chertow GM, Fan D, McCulloch CE, Hsu CY. Chronic kidney disease and the risks of death, cardiovascular events, and hospitalization. N Engl J Med. 2004;351(13):1296-305.

6. Xie X, Liu Y, Perkovic V, et al. Renin-angiotensin system inhibitors and kidney and cardiovascular outcomes in patients with CKD: a Bayesian network meta-analysis of randomized clinical trials. Am K Kidney Dis. 2016;67(5):728-41.

7. UK Renal Association. UK Renal Registry 18th Annual Report 2015. 2016. https://www.renalreg.org/wp-content/uploads/2015/01/web_book_07-04-16.pdf. Accessed 8 Dec 2016.

8. National Institute for Health and Care Excellence. Chronic kidney disease (stage 5): peritoneal dialysis. Clinical guideline [CG125]. 2011. https://www.nice.org.uk/guidance/cg125. Accessed 8 Dec 2016.

9. NHS Blood and Transplant. Annual report on kidney transplantation. Report for 2015/2016 (1 April 2006 – 31 March 2016). 2016. http://www.odt.nhs.uk/pdf/organ_specific_report_kidney_2016.pdf. Accessed 8 Dec 2016.

10. Gorodetskaya I, Zenios S, Mcculloch CE, et al. Health-related quality of life and estimates of utility in chronic kidney disease. Kidney Int. 2005;68(6):2801-8.

11. Lee AJ, Morgan CL, Conway P, Currie CJ. Characterisation and comparison of health-related quality of life for patients with renal failure. Curr Med Res Opin. 2005;21(11):1777-83.

12. Sullivan PW, Slejko JF, Sculpher MJ, Ghushchyan V. Catalogue of EQ-5D scores for the United Kingdom. Med Decis Making. 2011;31(6):800-4.

13. British Heart Foundation. Cardiovascular Disease Statistics 201. 2015. https://www.bhf.org.uk/publications/statistics/cvd-stats-2015. Accessed 8 Dec 2016.

14. Haacke C, Althaus A, Spottke A, et al. Long-term outcome after stroke evaluating health-related quality of life using utility measurements. Stroke. 2006;37(1):193-8.

15. Holland R, Rechel B, Stepien K, Harvey I, Brooksby I. Patients' self-assessed functional status in heart failure by New York Heart Association class: a prognostic predictor of hospitalizations, quality of life and death. J Card Fail. 2010;16(2):150-6.

16. Lacey EA, Musgrave RJ, Freeman JV, Tod AM, Scott P. Psychological morbidity after myocardial infarction in an area of deprivation in the UK: evaluation of a self-help package. Eur J Cardiovasc Nurs. 2004;3(3):219-24.

17. Göhler A, Geisler BP, Manne JM, et al. Utility Estimates for Decision-Analytic Modeling in Chronic Heart Failure – Health States Based on New York Heart Association Classes and Number of Rehospitalizations. Value Health. 2009;12(1):185-7.

18. Sennfalt K, Magnusson M, Carlsson P. Comparison of hemodialysis and peritoneal dialysis – a cost-utility analysis. Perit Dial Int. 2002;22(1):39-47.

19. National Institute for Health and Care Excellence. Chronic kidney disease in adults: assessment and management. Clinical guideline [CG182]. 2015. https://www.nice.org.uk/guidance/cg182. Accessed 28 July 2017.

20. Ponikowski P, Voors AA, Anker SD, et al. 2016 ESC Guidelines for the diagnosis and treatment of acute and chronic heart failure: The Task Force for the diagnosis and treatment of acute and chronic heart failure of the European Society of Cardiology (ESC). Eur Heart J. 2016;37(27):2129-200.

21. Haymarket Media Group Ltd. Monthly Index of Medical Specialities. 2017. http://www.mims.co.uk/. Accessed 16 Nov 2017.

22. Baboolal K, McEwan P, Sondhi S, et al. The cost of renal dialysis in a UK setting – a multicentre study. Nephrol Dial Transplant. 2008;23(6):1982-9.

23. Department of Health. NHS reference costs 2014 to 2015. 2015. https://www.gov.uk/government/publications/nhs-reference-costs-2014-to-2015. Accessed 8 Dec 2016.

24. Curtis L, Burns A. Personal Social Services Research Unit (PSSRU) Unit Costs of Health and Social Care 2015. 2015. https://www.pssru.ac.uk/pub/uc/uc2015/full.pdf. Accessed 8 Dec 2016.
