# Supplementary material for: The value of maintaining normokalaemia and enabling RAASi therapy in chronic kidney disease
Source: BMC Nephrol. 2019 Jan 31;20:31. doi: 10.1186/s12882-019-1228-y (PMC6357372; doi:10.1186/s12882-019-1228-y)
Supplement: Supplementary file 3 — Figure S1. Model-estimated cumulative events over five years, according to CKD stage (a–c), RAASi use (d–f) and incidence of hyperkalaemia (g–i). Figure S2. Impact of RAASi cost and efficacy (0–100% of base case values) on discounted lifetime per-patient costs, QALYs, life years and NMB at conventional WTP thresholds. (DOCX 453 kb) [file 12882_2019_1228_MOESM3_ESM.docx]

Figure S1 Model-estimated cumulative events per 100 patients over five years, according to CKD stage (a–c), RAASi use (d–f) and incidence of hyperkalaemia (g–i)


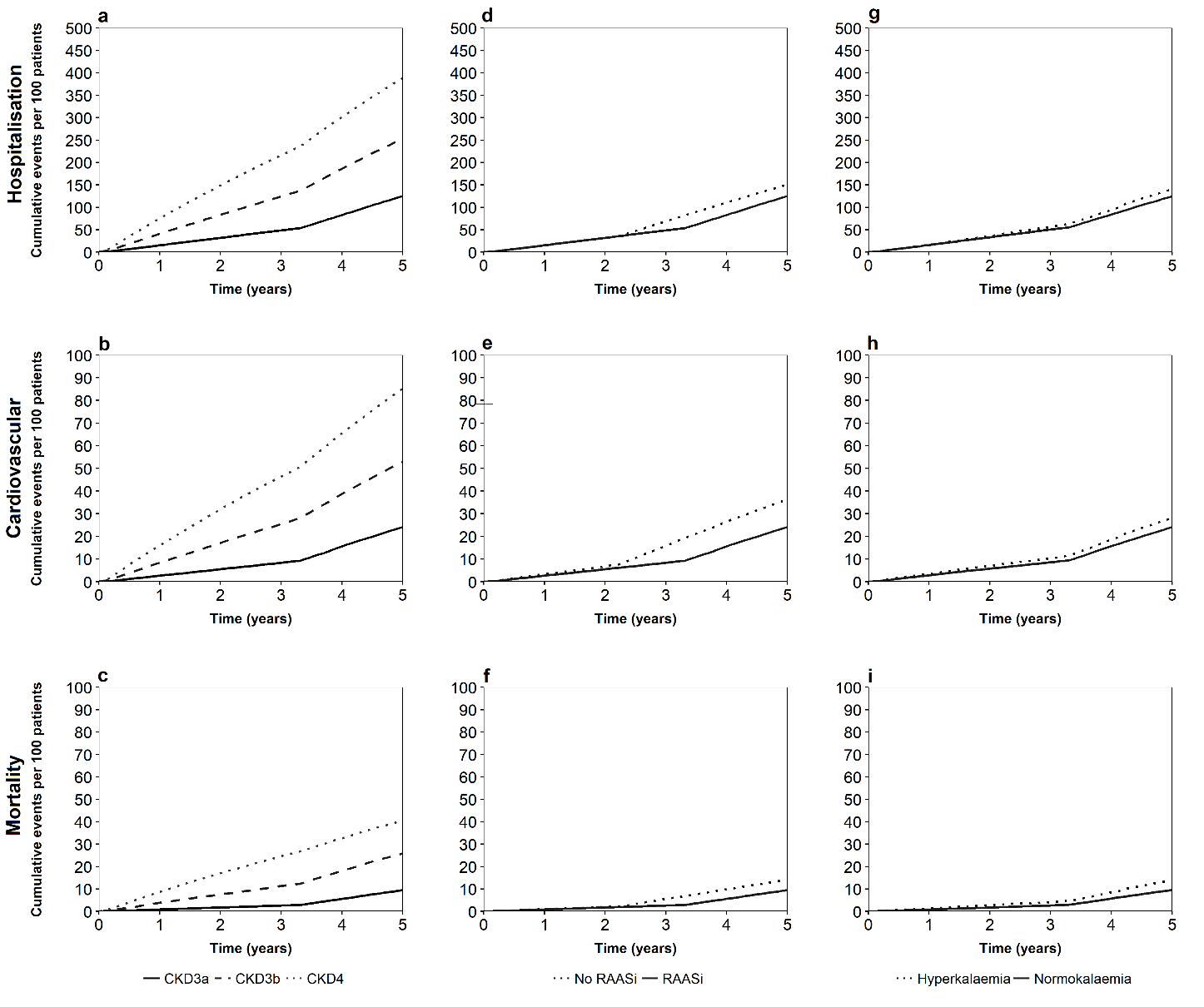


*CKD: chronic kidney disease; RAASi: renin-angiotensin-aldosterone system inhibitor;*

To aid interpretation of the relationship between key patient characteristics and modelled event rates, the cumulative number of mortality, cardiovascular and hospitalization events estimated by the model are presented, according to initial CKD severity (eGFR (mL/min/1.73m^2^): 52.5 (CKD3a), 37.5 (CKD3b), and 22.5 (CKD4)), renin-angiotensin-aldosterone system inhibitor (RAASi) use and serum potassium status (normokalaemia or hyperkalaemia).

All settings were as described in the base case scenario with RAASi use, unless otherwise specified. In the normokalaemia scenario constant potassium levels of 4.5 mEq/L were modelled; while in the hyperkalaemia scenario hyperkalaemia events (potassium ≥6 mEq/L) were modelled in two months per year. Simulated patients on RAASi progress to the next CKD stage at 3.4 years; while those not on RAASi progress at 2.4 years.

Figure S2 Impact of RAASi cost and efficacy (0–100% of base case values) on discounted lifetime per-patient costs, QALYs, life years and NMB at conventional WTP thresholds


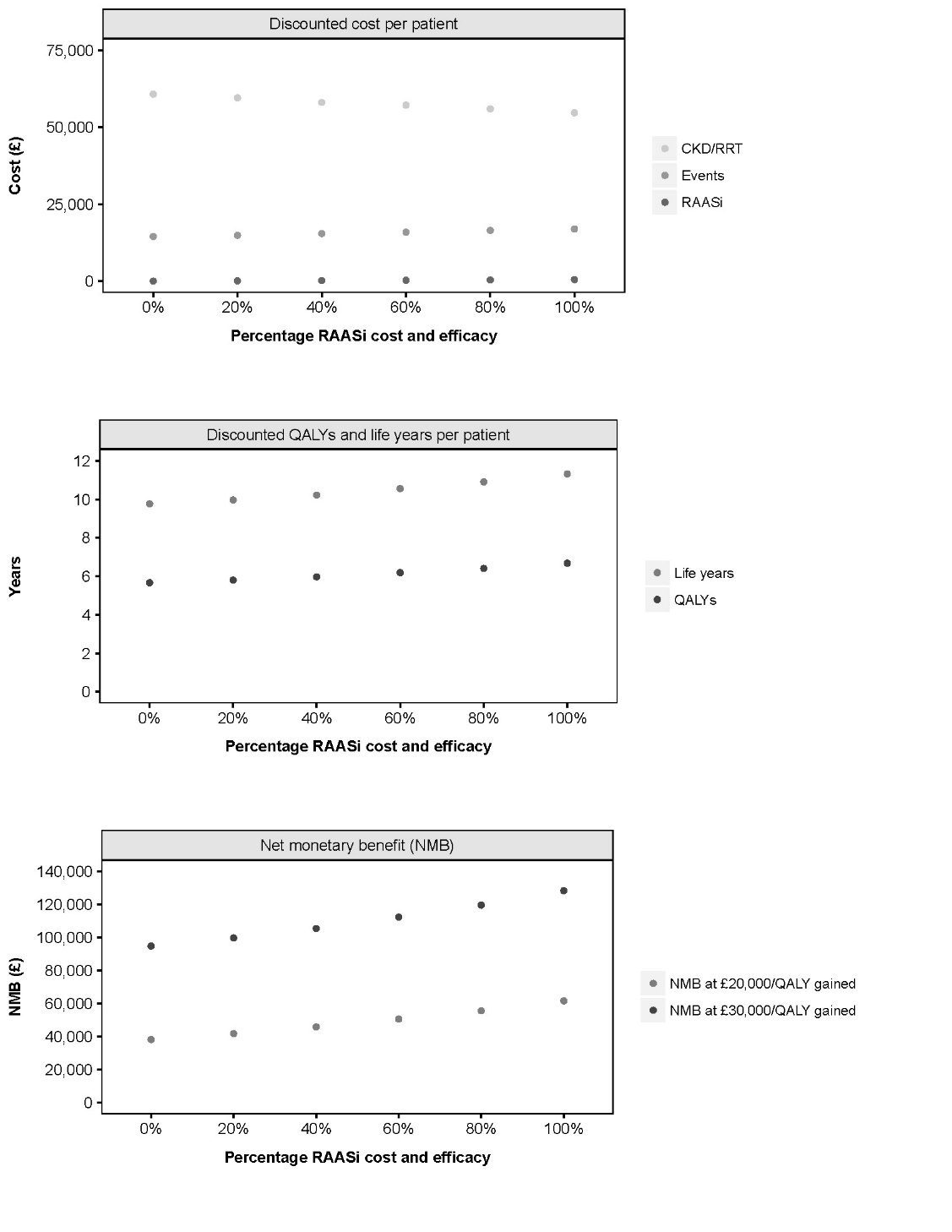


*CKD: chronic kidney disease; NMB: net monetary benefit; QALY: quality-adjusted life year; RAASi: renin-angiotensin-aldosterone system inhibitor; RRT: renal replacement therapy; WTP, willingness-to-pay*
